# On the Antagonism between Typhoid Fevers, Intermittents and Phthisis

**Published:** 1848-02

**Authors:** 


7. *On the Antagonism between Typhoid Fevers, Intermittents, and Phthisis.* By M. Boudin.

The following summary of M. Boudin's conclusions on this subject are given in a recent number of the *British and Foreign Review.* The facts are simple, and the sources from whence they are obtained sufficiently accurate to justify a reliance on the statements.

1. Those localities in which the producing cause of endemic intermittents thoroughly modify the constitution of man, are remarkable for the infrequency of pulmonary phthisis and typhoid fever. 2. The localities in which pulmonary phthisis and typhoid fever are particularly prevalent, are remarkable for the infrequency and mildness of intermittent fevers contracted on the spot. 3. The drying up of a marsh, or its conversion into a lake, diminishes or prevents intermittent fevers, but seems to dispose the organism to a new series of diseases, in which pulmonary phthisis and (according to the climate) typhoid fever are particularly prominent. 4. After a residence in a thoroughly marshy locality, an individual enjoys an immunity from typhoid fever, the degree and duration of which is in direct proportion, first, to the length of the previous residence; second, to the intensity of the fevers proper to the locality, considered under the two-fold relations of form and type; third, or, in other words, that a residence in a country of remittent and continued fevers, such as certain points of the coast of Algeria, and the centre of the marshy part of Brasse, is more prophylactic against the disease referred to, than, for example, a residence near the marshy embouchure of the Bievre, at Paris. 5. The conditions of latitude and longitude, and of height (above the sea) which limit the manifestation of marsh fevers, equally limit the curative or prophylactic influence of the marsh miasm. 6. Lastly, certain conditions of race, and possibly of sex, diminish the susceptibility of the system to the cause of marsh fevers, and in equal degree diminish the therapeutic influence of that cause.

The subject of which M. Boudin treats has considerable practical value in the distribution of troops, and in the hygeine of those predisposed to consumption.—*Lancet. From ibid.*

